# Neighborhood Greenspace and Socioeconomic Risk are Associated with Diabetes Risk at the Sub-neighborhood Scale: Results from the Prospective Urban and Rural Epidemiology (PURE) Study

**DOI:** 10.1007/s11524-022-00630-w

**Published:** 2022-05-12

**Authors:** Blake Byron Walker, Sebastian Tobias Brinkmann, Tim Große, Dominik Kremer, Nadine Schuurman, Perry Hystad, Sumathy Rangarajan, Koon Teo, Salim Yusuf, Scott A. Lear

**Affiliations:** 1 Community Health Environments and Social Terrains Lab, Institute for Geography, Friedrich-Alexander-Universität Erlangen-Nürnberg, Wetterkreuz 15, 91058 Erlangen, Germany; 2 Faculty of Health Sciences, Simon Fraser University, Burnaby, Canada; 3 Spatial Health Lab, College of Public Health and Human Sciences, Oregon State University, Corvallis, USA; 4 Population Health Research Institute, McMaster University, Hamilton, Canada; 5 Department of Geography, Simon Fraser University, Burnaby, Canada

**Keywords:** Diabetes, Greenspace, Socioeconomic status, GIS, Indices

## Abstract

Greenspace and socioeconomic status are known correlates of diabetes prevalence, but their combined effects at the sub-neighborhood scale are not yet known. This study derives, maps, and validates a combined socioeconomic/greenspace index of individual-level diabetes risk at the sub-neighborhood scale, without the need for clinical measurements. In two Canadian cities (Vancouver and Hamilton), we computed 4 greenspace variables from satellite imagery and extracted 11 socioeconomic variables from the Canadian census. We mapped 5125 participants from the Prospective Urban and Rural Epidemiology Study by their residential address and used age- and sex-dependent walking speeds to estimate individual exposure zones to local greenspace and socioeconomic characteristics, which were then entered into a principal component analysis to derive a novel diabetes risk index (DRI-GLUCoSE). We mapped index scores in both study areas and validated the index using fully adjusted logistic regression models to predict individual diabetes status. Model performance was then compared to other non-clinical diabetes risk indices from the literature. Diabetes prevalence among participants was 9.9%. The DRI-GLUCoSE index was a significant predictor of diabetes status, exhibiting a small non-significant attenuation with the inclusion of dietary and physical activity variables. The final models achieved a predictive accuracy of 75%, the highest among environmental risk models to date. Our combined index of local greenspace and socioeconomic factors demonstrates that the environmental component of diabetes risk is not sufficiently explained by diet and physical activity, and that increasing urban greenspace may be a suitable means of reducing the burden of diabetes at the community scale.

## Background

Type 2 diabetes is a multifactorial disease with foundations in genetic and behavioural risk factors. However, with the continuing increases in the prevalence of diabetes among countries of all economic strata, these factors do not comprehensively explain risk for diabetes and suggests societal and environmental factors are also involved, including the socioeconomic composition and geographical context of a neighborhood. This is reflected in the WHO’s 2016 statement underscoring the importance of socioeconomic conditions and healthy neighborhood environments as a focus area for prevention policy [1].

Previous studies have identified a higher prevalence of diabetes in subpopulations with low socioeconomic status (SES), most commonly measured using composite indices that capture diverse aspects of socioeconomic (dis)advantage [2, 3]. When measured at the individual- or household-level, socioeconomic risk factors for diabetes include income [4], educational attainment [5, 6], and occupational status [5]. Individual SES is not distributed randomly, and low and high SES individuals cluster together, which can be measured using area-based SES measures, for example, using census tracts. At the neighborhood scale, these measures represent the socioeconomic composition of an area and have been used to identify policy interventions targeting the socioeconomic component of disease risk. In public health planning and prevention programmes, area-based SES indices (e.g., the Vancouver Area Neighborhood Deprivation Index) have demonstrated immense value as correlates for a wide range of diseases, syndromes, and health conditions [7]. Even when controlling for individual- or household-level SES, area-based metrics often remain significant predictors, suggesting a social-environmental/neighborhood-scale compositional component to the effects of SES on health [5, 8, 9].

In addition to the socioeconomic composition of a neighborhood, the geographical/structural context is known to exhibit effects on health. Many recent studies have focussed on neighborhood-scale contextual characteristics relevant to diabetes risk, predominantly greenspace and walkability; both are known to be associated with a lower diabetes incidence/prevalence, as they provide enhanced opportunities for physical activity [10–15]. In addition, greenspace may capture other pathways important to diabetes risk, such as reducing air pollution exposures [16], decreasing stress [16], or increasing neighborhood social capital [17]. As such, policy and community-based initiatives to improve or enhance urban greenspace are often presented as measures for improving population health [1].

In many locations, the level of neighborhood greenspace is associated or correlated with SES and studies have attempted to disentangle the two and examine their effects in isolation using statistical adjustment [18]. However, we hypothesise that the health-related benefits of urban greenspace may, to a limited degree, mitigate the risk associated with socioeconomic deprivation. As such, area-level predictive models and risk indices should take not only the socioeconomic composition of a neighborhood into account, but also include contextual factors such as greenspace. The effects of neighborhood SES and greenspace are unlikely to be independent but rarely has the combined influence of these exposures been examined. This study therefore aims to examine the combined effects of local SES and walkable greenspace on diabetes risk to develop a novel, high-resolution geographical exposure model. We derived and validated a high-resolution, area-based Diabetes Risk Index of Green Land Use and Community Socioeconomic Environments (DRI-GLUCoSE) in two Canadian cities and their rural peripheries and mapped the results as follows.

## Methods

### Participant Data

The current cross-sectional study focusses on 5125 participants recruited between 2006 and 2009 in Hamilton and Vancouver, Canada, for whom we had robust address and community-level characteristics. These sites are part of the PURE study, which is an ongoing global cohort study investigating risk factor for chronic disease in 27 countries [19]. All participants provided informed consent and the study was approved by the local institution research ethics boards.

Individual variables collected using surveys included residential address, age, sex, household income range, tobacco and alcohol consumption, history of diagnosed diabetes, and history of diabetes medication use. Additionally, each participant’s diet was determined using the alternative healthy eating index (AHEI), a nine-component index on dietary choices and nutrient intake designed to assess dietary-based chronic disease risk [20], and daily physical activity metabolic equivalent of task (MET) scores were calculated from robust questionnaire instruments as described by Lear et al. [21]. Trained project staff conducted in-person measurements to derive body mass index (BMI, measured as a continuous variable) and waist-to-hip ratio (WHR).

Participants were classified as having diabetes (either type 1 or type 2) if they fulfilled any of the following criteria: self-reported a diagnosis of diabetes; current or past use of diabetes-specific medication; a fasting plasma glucose level greater than or equal to 7.0 mmol/l.

### Environmental Data

Data for Canadian census dissemination areas (DA, the smallest available census area with an average population of 400–800 residents) for the census year 2006 were acquired from Statistics Canada. The SES variables included in this analysis are listed in Table 1. Each DA was categorised as urban/suburban/rural using a previously validated, Canada-specific classification method based on active transportation and population density [7, 22, 23].

**Table 1:** Study participants and neighborhood characteristics by diabetes status

	No diabetes (*N* = 4616, 90.1%)	Diabetes (*N* = 509, 9.9%)	Total (*N* = 5125)	Bivariate odds ratio OR (95% CI, *p*-value)
City				
Hamilton	2307 (87.5%)	331 (12.5%)	2638	
Vancouver	2309 (92.8%)	178 (7.2%)	2487	0.54 (0.44–0.65, *p* < 0.001)
BMI				1.12 (1.11–1.14, *p* < 0.001)
Mean (SD)	27.3 (5.4)	32.1 (6.4)	27.8 (5.7)	
Median (Q1; Q3)	26.4 (23.8; 29.8)	30.9 (27.5; 35.7)	26.8 (24.0; 30.4)	
Waist to hip ratio				1.11 (1.10–1.12, *p* < 0.001)
Mean (SD)	85.2 (9.0)	93.6 (8.5)	86.0 (9.3)	
Median (Q1; Q3)	85.2 (78.6; 91.6)	94.2 (87.8; 99.6)	86.0 (79.3; 92.6)	
Obesity (WHR)				
Low and moderate	2787 (96.0%)	115 (4.0%)	2902	
High	1829 (82.3%)	394 (17.7%)	2223	5.22 (4.21–6.48, *p* < 0.001)
Age (years)				1.29 (1.23–1.36, *p* < 0.001)
Mean (SD)	52.7 (9.4)	56.9 (8.3)	53.1 (9.3)	
Median (Q1; Q3)	53.0 (45.0, 60.0)	58.0 (51.0, 63.0)	53.0 (46.0, 61.0)	
Sex				
Male	2076 (87.6%)	294 (12.4%)	2370	
Female	2540 (92.2%)	215 (7.8%)	2755	0.60 (0.50–0.72, *p* < 0.001)
Household income range				0.75 (0.71–0.80, *p* < 0.001)
> 90 k	1793 (93.3%)	129 (6.7%)	1922	
45 k–65 k	759 (89.8%)	86 (10.2%)	845	
30 k–45 k	564 (85.2%)	98 (14.8%)	662	
65 k–90 k	1010 (92.7%)	80 (7.3%)	1090	
20 k–30 k	304 (81.3%)	70 (18.7%)	374	
< 20 k	186 (80.2%)	46 (19.8%)	232	
AHEI score				0.97 (0.96–0.98, *p* < 0.001)
Mean (SD)	37.7 (10.0)	35.0 (9.5)	37.4 (9.9)	
Median (Q1; Q3)	37.6 (30.7, 44.8)	34.8 (28.1, 41.4)	37.3 (30.4, 44.5)	
Physical activity MET score				
< 525	1721 (87.3%)	250 (12.7%)	1971	
≥ 525	2065 (92.3%)	172 (7.7%)	2237	0.57 (0.47–0.70, *p* < 0.001)
Ever smoked				
No	2087 (92.3%)	174 (7.7%)	2261	
Yes	1699 (87.3%)	248 (12.7%)	2937	1.75 (1.43–2.15, *p* < 0.001)
Daily drinker				
≥ 1 drink/day	1157 (91.0%)	114 (9.0%)	1271	
< 1 drink/day	2629 (89.5%)	308 (10.5%)	2937	1.19 (0.95–1.49, *p* = 0.133)
Participants’ neighborhood socioeconomic status				
Neighborhood type				
Suburban/rural	3607 (89.9%)	405 (10.1%)	4012	
Urban	1009 (90.7%)	104 (9.3%)	1113	0.92 (0.73–1.15, *p* = 0.459)
Individual mean income (CAD/1000)				0.97 (0.96–0.98, *p* < 0.001)
Mean (SD)	37.9 (13.9)	34.3 (12.1)	37.5 (13.8)	
Median (Q1; Q3)	35.0 (29.0; 42.0)	32.0 (27.0; 38.0)	35.0 (29.0; 42.0)	
Household median income (CAD/1000)				0.99 (0.98–0.99, *p* < 0.001)
Mean (SD)	65.5 (21.5)	60.6 (20.5)	65.0 (21.4)	
Median (Q1; Q3)	61.0 (51.0; 77.0)	57.0 (45.0; 71.0)	61.0 (51.0; 76.0)	
Prevalence of low income (%)				1.04 (1.02–1.05, *p* < 0.001)
Mean (SD)	9.4 (6.9)	11.3 (7.6)	9.6 (7.0)	
Median (Q1; Q3)	8.1 (4.3; 13.7)	10.1 (5.2; 16.3)	8.2 (4.4; 14.0)	
Commute walking/bicycle (%)				0.98 (0.97–1.00, *p* = 0.027)
Mean (SD)	7.5 (6.9)	6.8 (5.8)	7.4 (6.8)	
Median (Q1; Q3)	5.1 (3.0; 9.9)	5.3 (3.2; 8.6)	5.2 (3.0; 9.7)	
Labour force participation rate (%)				0.98 (0.97–0.99, *p* < 0.001)
Mean (SD)	66.6 (7.7)	65.3 (7.6)	66.5 (7.7)	
Median (Q1; Q3)	66.8 (61.8; 71.7)	64.9 (60.7; 70.5)	66.6 (61.7; 71.6)	
Gov’t transfer payments (%)				1.08 (1.06–1.09, *p* < 0.001)
Mean (SD)	9.9 (5.2)	12.4 (6.3)	10.1 (5.4)	
Median (Q1; Q3)	8.8 (5.7; 13.0)	11.5 (7.2; 16.1)	9.0 (5.8; 13.3)	
Unemployment rate (%)				1.11 (1.08–1.15, *p* < 0.001)
Mean (SD)	5.5 (2.5)	6.3 (2.9)	5.6 (2.6)	
Median (Q1; Q3)	5.4 (3.8; 7.0)	5.9 (4.4; 7.7)	5.4 (3.8; 7.0)	
Lone parent families (%)				1.05 (1.04–1.07, *p* < 0.001)
Mean (SD)	14.8 (6.5)	17.3 (7.5)	15.0 (6.6)	
Median (Q1; Q3)	14.0 (10.9; 18.4)	16.1 (11.9; 21.8)	14.1 (11.0; 18.8)	
Education—no degree (%)				1.04 (1.03–1.05, *p* < 0.001)
Mean (SD)	19.6 (9.2)	23.7 (10.4)	20.0 (9.4)	
Median (Q1; Q3)	17.7 (12.8; 24.9)	21.0 (15.8; 31.6)	17.9 (13.1; 25.6)	
Private dwellings—owned (%)				1.00 (0.99–1.00, *p* = 0.222)
Mean (SD)	73.9 (17.8)	72.9 (17.6)	73.8 (17.7)	
Median (Q1; Q3)	77.1 (62.1; 88.5)	75.6 (61.7; 87.0)	77.0 (62.1; 88.4)	
Private dwellings—rented (%)				1.00 (1.00–1.01, *p* = 0.166)
Mean (SD)	25.3 (17.2)	26.4 (17.0)	25.4 (17.2)	
Median (Q1; Q3)	22.1 (11.2; 36.9)	24.0 (12.9; 37.7)	22.4 (11.4; 36.9)	
Participants’ neighborhood greenspace: Normalized Difference Vegetation Index (NDVI); OR per 0.1 NDVI-unit increase				
NDVI—median				0.68 (0.61–0.77, *p* < 0.001)
Mean (SD)	0.341 (0.089)	0.314 (0.084)	0.338 (0.089)	
Median (Q1; Q3)	0.336 (0.291; 0.379)	0.313 (0.266; 0.360)	0.333 (0.288; 0.377)	
NDVI—standard deviation				0.79 (0.55–1.13, *p* = 0.201)
Mean (SD)	0.090 (0.026)	0.088 (0.024)	0.090 (0.026)	
Median (Q1; Q3)	0.086 (0.071; 0.105)	0.084 (0.072; 0.100)	0.086 (0.071; 0.105)	
NDVI—Min				0.75 (0.67–0.83, *p* < 0.001)
Mean (SD)	0.206 (0.097)	0.182 (0.086)	0.204 (0.096)	
Median (Q1; Q3)	0.202 (0.135; 0.262)	0.173 (0.118; 0.243)	0.198 (0.134; 0.260)	
NDVI—Max				0.69 (0.62–0.77, *p* < 0.001)
Mean (SD)	0.495 (0.093)	0.465 (0.093)	0.492 (0.093)	
Median (Q1; Q3)	0.484 (0.435; 0.546)	0.459 (0.401; 0.514)	0.482 (0.432; 0.543)	

The Normalized Difference Vegetation Index (NDVI) provides a greenspace metric often used in health research [24]. Derived from satellite imagery, higher NDVI values indicate a greater intensity of greenness. We acquired cloud-free LANDSAT 5 satellite images for Hamilton (09.05.2006) and Vancouver (23.07.2006) through the United States Geological Survey’s EarthExplorer platform. Both images were preprocessed and NDVI scores were calculated and mapped as 30-m pixels (native resolution) in the study areas.

### Spatial Modelling

Data preparation and analysis were conducted using R (v.4), and mapping was completed using QGIS (v.3.10) on a Linux system (AMD Ryzen 9 3900 × CPU, 64 GB DDR4). All code including detailed documentation are available at https://github.com/CHEST-Lab/DRIGLUCoSE.

**Fig. 1:**
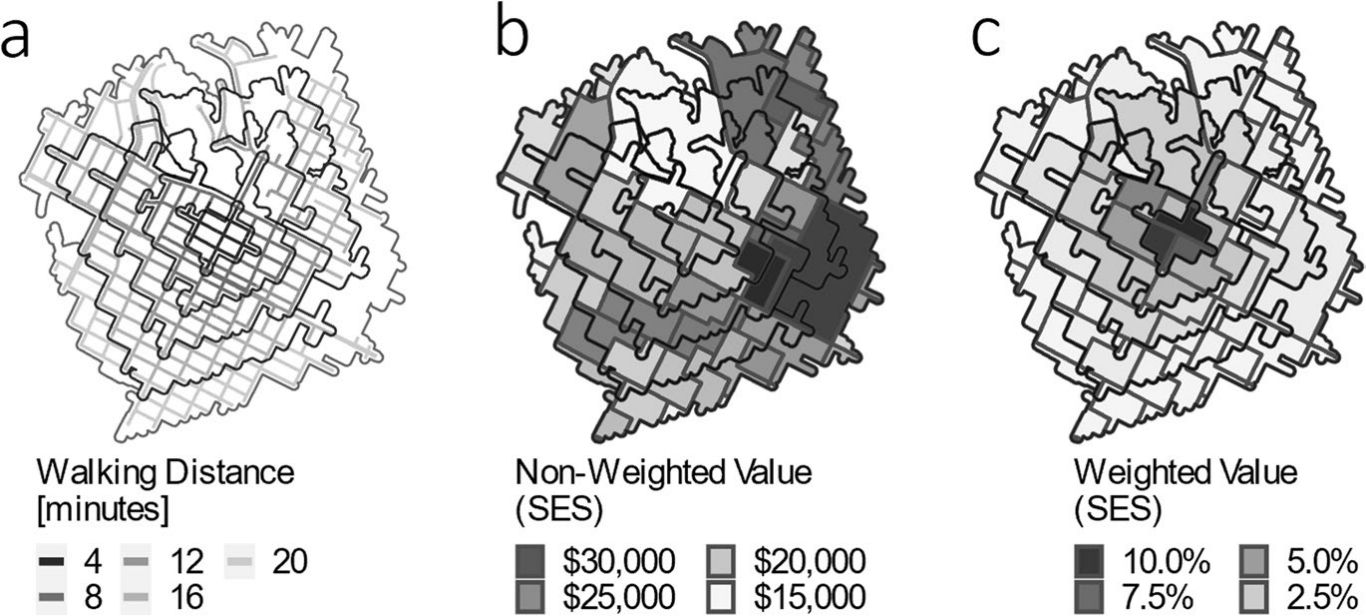
Spatial weighting procedure to account for diminishing effects of distance in which (**a**) a road network was used to compute isochrones, (**b**) an unweighted variable is mapped over the network, and (**c**) a distance-decay weighting scheme is applied to the variable

In order to estimate each participant’s potential exposures to greenspace and local SES, we mapped age- and sex-specific walkable buffer zones around each individual’s residential address using street and path networks derived from OpenStreetMap data (Fig. 1). Using age- and sex-specific walking speeds (average male–female difference = 0.13 km/h; [25]), we then mapped each participant’s walkable areas in 2-min increments from their residential address, with a maximum walking time of 20 min. The 20-min maximum parameter was derived through sensitivity analysis, in which walking areas were calculated in 2-min intervals and iteratively entered into the successive statistical models described below. The 20-min parameter was selected as it (i) featured the highest predictive performance, (ii) is a heuristically realistic representation of movement patterns in the city, in that it adequately captures activity spaces of persons in the study (this was ascertained through informal discussion with residents of the study area, in which several of the authors reside), and (iii) the 20-min zone also approximates the radius of structurally homogenous spaces/neighborhoods in the study area and may therefore be the most suitable proxy for composition of the built environment. Each participant was thereby assigned ten concentric walking zones from 0 to 20 min walking time (Fig. 1a). The concentric walking zones were then overlaid on the SES and greenspace data described above (Fig. 1b). A logit weighting function was applied to each buffer zone’s mean distance to derive distance-based variable weights, such that the estimated effect of an SES or greenspace variable decreases as distance from the home increases (Fig. 1c). A logit function was selected as it heuristically approximates a suitable distance-decay function [26] and various parameterisations were assessed through sensitivity analysis. The zone-distance-weighted mean of each of the 15 SES variables and 4 greenspace variables was then assigned to each participant for index derivation. Equations and documented code are provided on our GitHub page, linked above.

In order to increase the amount of built environment information contained in the model, we calculated 4 separate metrics from the NDVI data: median NDVI score; standard deviation; and 95^th^ and 5^th^ percentiles. These were selected as they respectively represent overall local greenspace levels, variability in the amount and intensity of greenspace within each walking zone, the intensity of the most green areas (*P*_95_) that may serve as local attractants or forested areas within a walking zone, and non-green areas (*P*_5_) that are characteristic of a dense urban or industrial built environment (greyspace) and bare land, hypothesised to exhibit negative effects.

### Index Derivation

Using participant data and residential address locations, we first derived the index as follows: in a subsequent step, we calculated and mapped the index across both study areas.

In order to manage class imbalance (i.e., ratio of diabetes to non-diabetes participants) for the index derivation [27], we used a combination of undersampling and the SMOTE algorithm [28] to iteratively derive model calibration sets, as described in our code/documentation. To derive the DRI-GLUCoSE index, we used principal component analysis (PCA) [29–32]. The 11 SES and 4 greenspace variables listed in Table 1 were normalised and tested for non-sphericity and suitability using Bartlett’s test and for sampling adequacy using Kaiser–Meyer–Olkin before being entered into a PCA model. The resulting loadings from the first component were then applied as variable weights for the 15 input variables, the sum of which was then rescaled to a range of − 1, 1 and taken as the participant DRI-GLUCoSE score. The 4 SES variables with negligible Eigenvalues or absent from the first component were removed, as these had no detectable influence on model accuracy, resulting in a final index with 11 SES variables and 4 greenspace variables. The index is scaled such that high index values correspond with socially deprived areas and low greenspace.

For model comparison, we derived three variants of the index: (a) the combined DRI-GLUCoSE index described above; (b) a variant using only the SES variables; (c) a variant only using the greenspace variables. These are described in more detail on our GitHub page (linked above).

In order to map index scores across both study areas, we first removed non-residential land use from the maps, then overlaid a 50-m pixel grid. The 50-m parameter was selected to achieve a balance between computational load and spatial precision following sensitivity analysis of variable pixel sizes. From the centre of each 50-m pixel, we assume a standard participant with a 20-min walking zone and a fixed walking speed of 4.7 km/h [25]. The DRI-GLUCoSE index scores for each pixel were then calculated using the weighting procedure described above. This resulted in a DRI-GLUCoSE index map for both study areas.

### Statistical Modelling and Validation

Using individual-level diabetes as a binary response variable, three sets of logistic regression models were run: (a) bivariate models with the DRI-GLUCoSE index and all control variables (age, sex, BMI, household income, urban/rural household, AHEI, recreational physical activity, smoking status, and alcohol consumption); (b) semi-adjusted multivariable models using the DRI-GLUCoSE index, age, sex, BMI, household income, and urbanicity; and (c) fully adjusted multivariable models using all variables from model B, plus tobacco and alcohol consumption, diet, and physical activity. For comparison, we also computed a series of bivariate and (semi-/fully-)adjusted models using various combinations of the 4 greenspace variables and the univariate predictors without PCA, including testing for interaction effects between greenspace and SES predictors. Odds ratios (OR) with 95% confidence intervals (CI) and *p*-values were reported for all models. Experimental models and models using WHR as the adiposity measure are reported in the online documentation.

To assess model performance and test for overfitting, we randomly split the data into training (80%) and testing (20%) subsets, preserving the within-group diabetes prevalence. In-sample statistics (*j*-index, sensitivity, specificity, AROC, and accuracy) of the training subset were calculated through tenfold cross-validation and compared to the out-of-sample statistics of the testing subset.

## Results

### Sample Characteristics

Of the total 5125 participants included in this analysis, 10% were classified as having diabetes (Table 1). The mean BMI and WHR were 27.8 kg/m^2^ and 0.86, respectively, and participant median age was 53 years (IQR 46, 61) at the time of data collection. Approximately 28% of participants reported an average of more than one unit of alcohol consumed per day, and 58.8% of household annual incomes were above CAD $65,000. Participants were equally divided between the two cities, with 78% of participants residing in suburban or rural neighborhoods.

### DRI-GLUCoSE Index

Bartlett’s test results confirmed strong non-sphericity (*p* < 0.001) of the predictor variables, and Kaiser–Meyer–Olkin returned an overall measure of sampling adequacy of 0.74, indicating a reasonable degree of suitability. The PCA results indicate that 49.3% of variance was explained in the first component, whose factor loadings for the derived index are shown in Table 2. The remaining components respectively explained 14.5%, 9.6%, 6.8%, and 5.2% of variance and were discarded. The factor loadings for the first component were then used as variable weights and entered a weighted linear combination to derive index scores.

**Table 2:** PCA results. Factor loadings in PC1 correspond to variable weights for the DRI-GLUCoSE index

Variable	Factor loadings (PC1)	Factor loadings (PC2)
Government transfer payments	− 0.35	0.19
Lone parent families	− 0.32	0.07
Household median income	0.31	0.18
Prevalence of low income	− 0.30	− 0.09
Unemployment rate	− 0.29	0.15
Education—no degree	− 0.29	0.35
NDVI—median	0.26	− 0.12
NDVI—5th percentile	0.26	0.07
NDVI—95th percentile	0.25	− 0.29
Individual mean income	0.24	− 0.04
Private dwellings—owned	0.24	0.45
Private dwellings—rented	− 0.24	− 0.44
Labour force participation rate	0.12	− 0.08
Commute active	− 0.10	− 0.32
NDVI—standard deviation	− 0.02	− 0.41
**Total variance explained**	**49.3%**	**14.5%**

Bartlett’s test for sphericity *p* < 0.001

Kaiser–Meyer–Olkin test for measure of sampling adequacy = 0.74

**Fig. 2:**
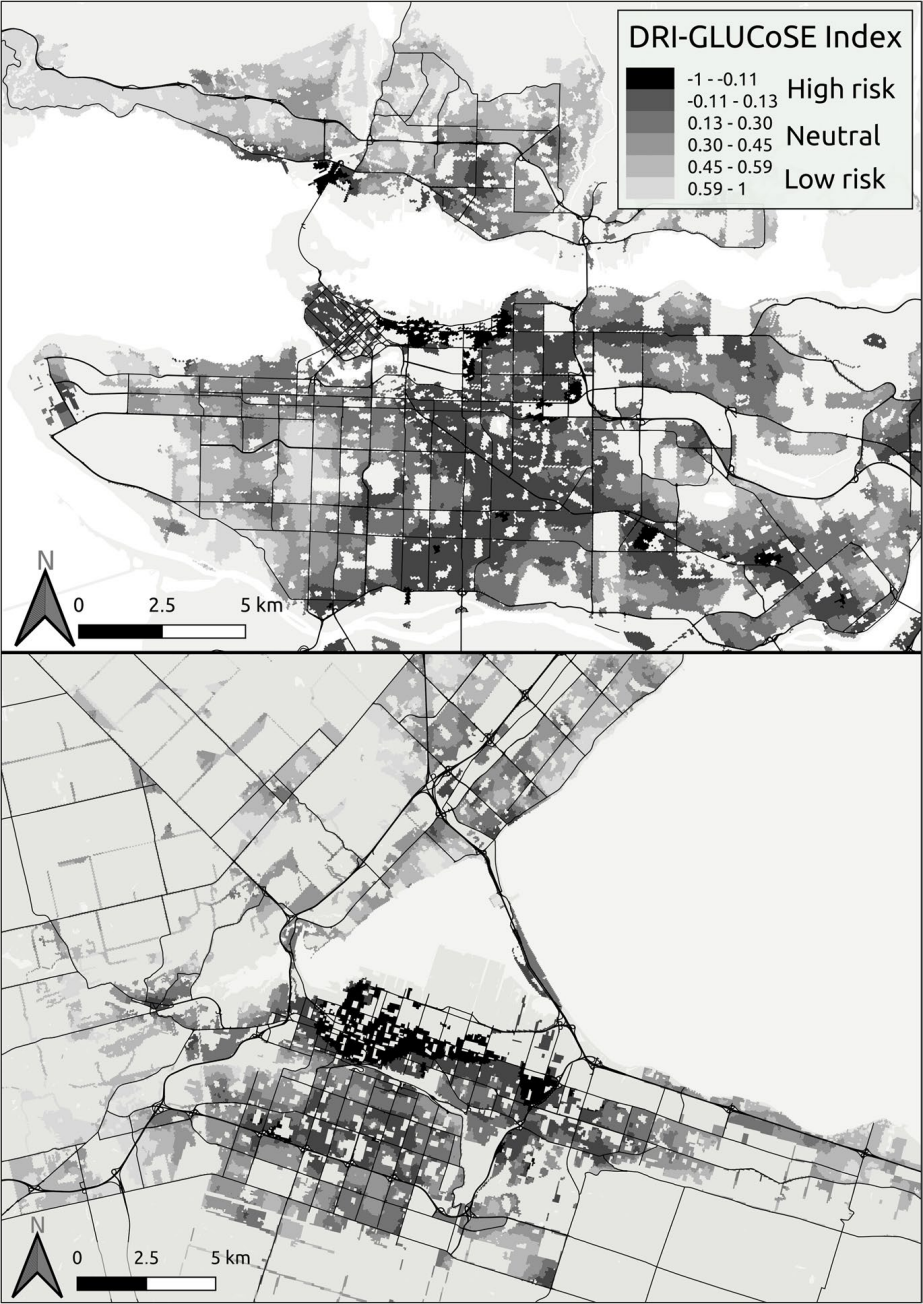
DRI-GLUCoSE scores for Vancouver (top) and Hamilton (bottom), ranging from low risk (light) to high risk areas (dark)

The mapped index values (Fig. 2) highlight areas where the local SES and greenspace conditions predict a higher (dark) or lower (light) diabetes risk.

### Regression Models

**Fig. 3:**
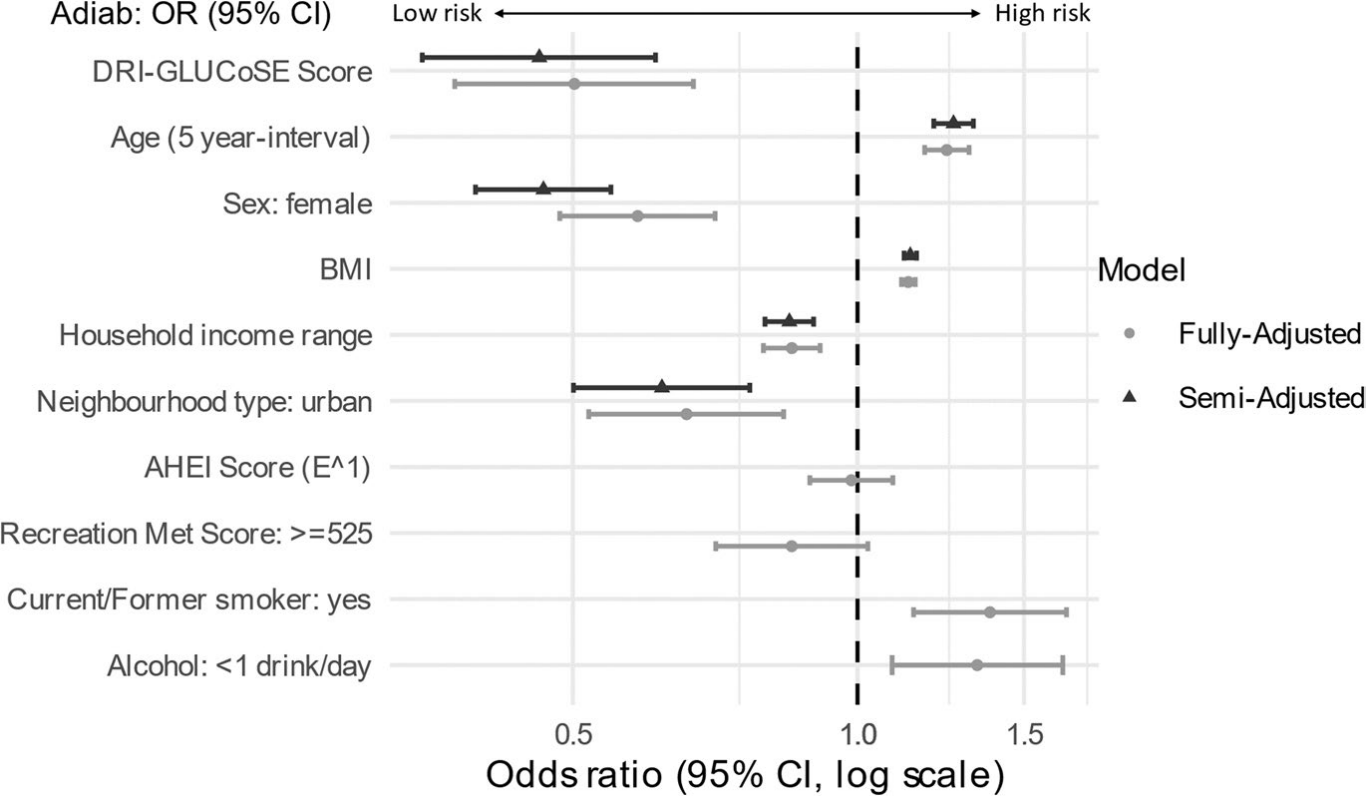
Forest plot showing significant effects for both semi-adjusted and fully adjusted multivariable logistic models. DRI-GLUCoSE, Diabetes Risk Index-Green Land Use and Community Socioeconomic Environments; BMI, body mass index; AHEI, Alternative Healthy Eating Score

The results of the logistic regression models based on the testing subset are shown in Fig. 3. Participants’ local DRI-GLUCoSE score exhibited a consistent positive association across all models. A small, non-significant attenuation of this effect was observed in the fully adjusted models, where individual-level lifestyle factors were included (diet, physical activity, smoking, and alcohol consumption). Increased odds of diabetes were observed for obese participants, current/former smokers, and persons who consume an average of less than one unit of alcohol per day. A lower risk is observed for urban residence, higher household incomes, and physical activity scores, but healthy eating score was non-significant (odds ratios shown in online appendix: https://github.com/CHEST-Lab/DRIGLUCoSE).

The multivariable models’ accuracies were 75% for the semi-adjusted model (Youden Index = 0.41, sensitivity = 0.78, specificity = 0.62, AROC = 0.76) and 75% for the fully adjusted model (Youden Index = 0.41, sensitivity = 0.75, specificity = 0.71, AROC = 0.78). Model diagnostics indicated no overfitting. Experimental models based on alternative configurations of greenspace variables, univariate (non-PCA) predictor variables, and interactions between greenspace and SES predictors consistently featured lower accuracy and/or featured poor model fit.

The final models were compared to identically parameterised logistic models using two additional variants of the DRI-GLUCoSE index: SES only and greenspace only. The combined SES and greenspace model achieved the highest prediction accuracy of 75%, compared to 72% for SES only (see Appendix).

## Conclusions

Numerous studies since the 1970s have demonstrated the power of SES for identifying and predicting health risks and outcomes. In this study, we augmented the utility of SES indices by including a novel model for greenspace, which is known to be a strong predictor for physical activity and positive health status/outcomes. Our regression model results indicate the DRI-GLUCoSE index is significantly associated with reduced odds of individual-level diabetes risk in two test cities in Canada, and its use of both greenspace and SES provides the strongest environmental risk model to date (75% prediction accuracy) for predicting individual diabetes risk using only non-invasive data. These results indicate that one’s diabetes risk is associated with their local neighborhood greenspace and SES and contribute to a growing recognition of geographical factors as important predictors of disease risk.

The index results are concurrent with the literature, in that higher neighborhood SES and local greenspace exhibit a protective effect [2, 5, 11, 12, 14, 15]. In terms of urban/regional planning policy and diabetes prevention, low SES, unhealthy food environments, and lack of infrastructure for physical activity have been underscored as key priorities [1]. Markevych et al. [33] and Astell-Burt et al. [15] categorized the mechanisms linking greenspace to health benefits into three domains: mitigation (e.g., better air quality); restoration (e.g., stress recovery); and instoration (e.g., promotion of physical activity) and emphasise the interconnectedness of mediation pathways, which has not yet been thoroughly addressed in the literature [34]. Our modelling results largely correspond with previous studies, in that low household income [4] and tobacco use [2, 35] were significant risk factors. Urban place of residence was associated with decreased diabetes odds in our models, as also reported by Basiak et al. [36], but contrary to Dagenais et al. [6], who observed elevated diabetes risk in urban areas. Recreational physical activity [6, 37] and healthy eating [38] exhibited a minor attenuation in the effect of the DRI-GLUCoSE index on diabetes risk. Also observed was an association between low/no alcohol consumption and increased diabetes odds, a result concordant with the literature [2, 39] and likely explained by patients being advised against alcohol consumption following a diabetes diagnosis [2, 36].

By accounting for environmental characteristics in the DRI-GLUCoSE index, we observed strong predictive performance based primarily on environmental characteristics. Other diabetes risk indices have been developed using a larger number of non-clinical participant-level variables and no environmental factors and have achieved similar predictive performance with an AROC of 0.78 [8] and 0.745 [40]. Within similar accuracy, the DRI-GLUCoSE index provides a free and easy-to-use risk estimation and mapping tool for estimating the combined effects of SES and greenspace on diabetes risk at the sub-neighborhood scale without the need for clinical measurements or individual-level data. The required socioeconomic data and satellite imagery are freely available for most regions of the globe and require minimal pre-processing, and the index calculation tool with documentation is freely available via GitHub, enabling public health analysts and researchers to calculate and map the index in the region of their choice. By mapping areas of interest using DRI-GLUCoSE, zones with a higher potential risk can be identified and targeted for urban renewal policy, diabetes prevention, patient counselling, and health services planning. However, it is important to note that the effect size exhibited by greenspace characteristics is significantly smaller than those of local and household SES and individual-level risk factors such as age, sex, and modifiable risk. The inclusion of greenspace to the SES-derived index only resulted in a 3% increase in overall accuracy in the fully adjusted models. This is indicative of (i) the relatively weak effects exhibited by local greenspace, and (ii) the importance of health-related behaviours in mitigating diabetes risk, regardless of geographical setting.

This study uses participant data that did not differentiate between type 1 and type 2 diabetes; given that the environmental effect on type 1 diabetes is likely to be low, it may be that our index and models underestimate the associations observed. While this study benefited from using two cities and their surrounding regions, both study areas are located in a high-income global region, and the index and modelling results may therefore not be generalisable to other global regions. Further research will focus on index refinement for middle- and low-income countries. Our study is limited by an exclusive focus on participants’ place of residence as their primary exposure region. Importantly, this study used a cross-sectional design, preventing any inference of causality. Finally, despite being demographically representative, the study cohort may exhibit some selection bias, as participation was voluntary. However, as the logistic models presented herein are similar to others from the literature, we believe these results to be adequately representative for the study population. Our results provide strong evidence for an environmental component to diabetes risk that is not accounted for by the selected covariates, but a dedicated study design to analyse the potential roles of, e.g., the food environment and air/noise/light pollution, may elucidate these effects in more detail.

The DRI-GLUCoSE index differs in that neighborhoods are defined not by administrative boundaries, rather, by age- and sex-specific walking zones. While this novel method for estimating exposure to greenspace and socioeconomic settings provides a more realistic representation of a person’s activity space, it is important to note that the downscaling of census data to a higher spatial resolution suffers from the assumption of demographic and socioeconomic homogeneity within the census units (i.e., the attributes of a census dissemination area are assigned to each individual resident within it, regardless of whether they reside in the centre of that area or on the border). So while this technique sought to mitigate facets of the ecological fallacy induced through spatial containerisation and boundary effects, it is not able to overcome these limitations entirely.

In this study, we derived and validated the DRI-GLUCoSE index as a high-resolution tool for quantifying and mapping the combined socioeconomic and greenspace component of local diabetes risk using epidemiological cohort data in two Canadian cities. The index remained a significant predictor of diabetes risk after controlling for individual-level modifiable risk factors. In the absence of individual-level data, neighborhood-level indices like DRI-GLUCoSE can provide a useful means for identifying areas of higher environmental risk. This is invaluable for planning prevention policy, designing healthy neighborhoods, and targeting patient counselling guidelines.
